# CAR-NK cell therapy combined with checkpoint inhibition induces an NKT cell response in glioblastoma

**DOI:** 10.1038/s41416-025-02977-8

**Published:** 2025-03-18

**Authors:** F. Strassheimer, P. Elleringmann, G. Ludmirski, B. Roller, J. Macas, T. Alekseeva, P. Cakmak, B. Aliraj, H. Krenzlin, M. C. Demes, I. C. Mildenberger, T. Tonn, K. J. Weber, Y. Reiss, K. H. Plate, A. Weigert, W. S. Wels, J. P. Steinbach, M. C. Burger

**Affiliations:** 1 Goethe University, Dr. Senckenberg Institute of Neurooncology, Goethe University Hospital, Frankfurt, Germany; 2 Frankfurt Cancer Institute (FCI), Frankfurt, Germany; 3 German Cancer Consortium (DKTK) and German Cancer Research Center (DKFZ), Heidelberg, Germany; 4 Goethe University, Institute of Neurology (Edinger Institute), Goethe University Hospital, Frankfurt, Germany; 5 Georg-Speyer-Haus, Institute for Tumor Biology and Experimental Therapy, Frankfurt, Germany; 6 Goethe University, Institute of Biochemistry I, Faculty of Medicine, Frankfurt, Germany; 7 University Medical Center Mainz, Department of Neurosurgery, Mainz, Germany; 8 Goethe University, Dr. Senckenberg Institute of Pathology, Goethe University Hospital, Frankfurt, Germany; 9 Department of Neurology, Medical Faculty Mannheim, University of Heidelberg, Mannheim, Germany; 10 Institute for Transfusion Medicine and Immunohematology, German Red Cross Blood Donation Service Baden-Württemberg-Hessen and Goethe University Hospital, Frankfurt, Germany

**Keywords:** Tumour immunology, Cancer microenvironment

## Abstract

**Background:**

Glioblastoma is the most aggressive primary brain tumor with limited efficacy of established therapies, and a pronounced immunosuppressive tumor microenvironment. Targeting HER2 with local immunotherapy allows for high tumor specificity in the brain with physiologically very low expression. Monotherapy with CAR-NK cells targeted against HER2 has previously shown efficacy in medium-sized GL261/HER2 tumors.

**Methods:**

Advanced GL261/HER2 tumors were treated by local CAR-NK cell injection combined with systemic anti-PD-1 checkpoint blockade. Tumor growth and survival were monitored. In-depth characterization of the microenvironment was performed by multiplex immune fluorescence, spectral flow cytometry and RNAseq.

**Results:**

Untreated GL261/HER2 tumors were characterized by local immunosuppression and high PD-L1 expression. Combined treatment with NK-92/5.28.z and systemic anti-PD-1 induced robust anti-tumor response and long-term survival. Multiplex immunofluorescence and spectral flow cytometry showed increased CD4^+^ T cell infiltration in mice treated with CAR-NK cell and anti-PD-1 combination therapy. A cluster of T cells specifically emerging in the combination therapy group expressed markers of NKT cells, which was further verified by immunofluorescence staining.

**Conclusion:**

The combination therapy reverted the immunosuppressive tumor microenvironment with increased T and NKT cell infiltration. This resulted in successful treatment of advanced orthotopic tumors refractory to CAR-NK cell monotherapy.

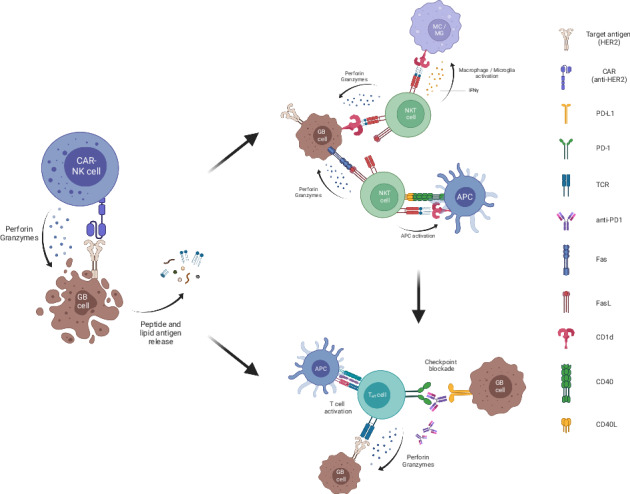

## Background

Glioblastoma (GB) is the most common and aggressive primary brain tumor in adults. Despite recent advances in the treatment of several other cancer entities, therapy of GB patients still relies on a multimodal regimen with surgical resection followed by radio- and chemotherapy and has not changed for more than a decade. However, relapse of tumors is practically inevitable, and the median overall survival reaches only around 15 months [1]. In contrast, in several other cancer indications the clinical development of immunotherapeutic approaches, especially immune checkpoint therapy (ICT) and adoptive cell therapy (e.g., CAR-T cell therapy), has shown promising results [2].

Checkpoint blockade of PD-1 or CTLA-4 has a considerable effect in malignancies like melanoma or NSCLC [2, 3]. Malignant cells frequently upregulate checkpoint molecules, like PD-1 or CTLA-4, to inhibit and evade the T-cell mediated immune response directed against the tumor. A similar effect was first described in chronic viral infections as a mechanism of immune regulation to prevent overarching immune responses [4]. Upon chronic antigen presentation, T cells frequently upregulate PD-1, which restricts their activation and can ultimately lead to terminal exhaustion. The phenotype of terminally exhausted T cells is mainly characterized by strong upregulation of PD-1, Lag-3, and Tim-3 [4]. However, Miller et al. [5] showed that exhausted tumor-infiltrating T cells still can be activated by checkpoint inhibition and then release proinflammatory cytokines like IFNγ and TNFα in vitro. Therefore, blockade of checkpoint receptors via antibody binding might disrupt the negative interaction of PD-1 and PD-L1 in vivo and increase T-cell activity.

So far clinical trials exploring immune checkpoint monotherapy in glioblastoma have been largely disappointing [6, 7]. Several studies have shown that glioblastomas are “immunologically cold” tumors with only few lymphocytes infiltrating. GB is characterized by a predominant infiltration of macrophages and microglia, with a rather low infiltration of T cells [8]. The immunosuppressive tumor microenvironment with few activated T cells contributes to resistance towards immunotherapy. The blood-brain barrier (BBB) impedes circulating immune cells from trafficking into the tumor site, which further restricts the intratumoral immune reaction by the failure of immune cell attraction into the tumor tissue. Immune checkpoint monotherapy seems not to be able to overcome such obstacles typical for glioblastoma, and to induce a clinically relevant immune reaction [9]. Interestingly, Cloughesy et al. [10] found that neoadjuvant PD-1 blockade with pembrolizumab increased both the immune response targeted against the tumor and overall survival as compared to adjuvant pembrolizumab therapy. One possible explanation for the responses observed in the neoadjuvant setting is the reduction of tumor mass after initiation of checkpoint blockade, diminishing the local immunosuppression and enabling the induction of a productive anti-tumor immune reaction by ICT.

Adoptive cellular therapy with chimeric antigen receptor (CAR)-engineered effector cells has so far mostly been explored in clinical trials with CAR-T cells targeting different tumor entities. CARs consist primarily of a single-chain fragment variable antibody, which is directed against a target molecule, linked to a CD3*ζ* or FcεRI*γ* signaling domain. Such first-generation CAR constructs have been optimized to include costimulatory protein domains, typically derived from CD28 and/or 4-1BB [11]. T cells carrying these second-generation CARs targeting CD19 or BCMA showed potent activity and are now clinical standard of care for several haematological malignancies [12]. CAR-T cell therapies targeting IL-13R*α*2, EGFRvIII and HER2 (ErbB2) have been explored in patients suffering from recurrent glioblastoma and resulted in acceptable safety with some signs of clinical activity [13–15].

Recently, CAR approaches based on natural killer (NK) cells as genetically modified effector cells have gained increased interest due to several potential advantages over CAR-T cells [16]. Manufacturing of CAR-T cells from patient-derived peripheral blood cells is costly and time-consuming. Moreover, transduction efficiency of viral CAR-vectors can vary significantly leading to inconsistent activity of the product [17]. Similar to CAR-T cells, CAR-NK cells can be generated through transduction of patient’s peripheral blood or iPSC-derived NK cells. However, a promising alternative to primary cells is the generation of CAR-NK cells as “off-the-shelf” product from cell lines like NK-92. This is a continuously growing cell line derived from a non-Hodgkin´s lymphoma patient that maintains many functional characteristics of activated NK cells. NK-92 has already been shown to be safe in four clinical trials without inducing graft-vs.-host disease [18–21]. Moreover, several clinical trials with CAR-NK-92 have already been conducted or are currently ongoing [22, 23]. Also, NK cells are not restricted to the interaction between T cell receptor and major histocompatibility complex proteins. The frequently observed downregulation of MHC molecules on tumor cells as a possible escape mechanism is not limiting NK cell activity due to their MHC-independent cytotoxicity. Furthermore, NK cells are in principle able to induce antibody-dependent cytotoxicity (ADCC) through CD16 and thus not limited to antigen-dependent cytolytic CAR activity. While NK-92 and its CAR derivates however do not express CD16 and therefore lack the ability to induce ADCC, they also do not express most inhibitory NK cell receptors except KIR2DL4, ILT-2 and NKG2A and have therefore a rather activated phenotype [16, 24].

Molecular profiling of glioblastoma cells has shown that up to 80% of GBs express HER2 [25], while the expression level in the surrounding brain is rather low. Furthermore, expression of HER2 is more stable over time than EGFRvIII, which is lost in around 50% at tumor recurrence [26, 27]. Thus, HER2 is an attractive target for CAR-mediated adoptive cellular therapy in GB patients. Introducing CAR-NK cell-directed therapy against HER2, Uherek et al. [28] and later Schönfeld et al. [29] engineered the CAR-NK cell line NK-92/5.28.z derived from NK-92, which was transduced with a CAR targeted against HER2. The respective second-generation CAR construct consists of the single chain fragment variable (scFv) FRP5 targeted against HER2, a CD28 transmembrane domain and the intracellular CD3*ζ* signaling domain. HER2 was also evaluated as a target in a clinical trial exploring CAR-T cells transduced with a very similar CAR for glioblastoma relapse therapy [13]. In vitro activity of NK-92/5.28.z has been shown against several HER2-positive glioblastoma cell lines, including primary glioblastoma stem cell cultures [27]. Also, NK-92/5.28.z cells were effective in several in vivo models including orthotopic glioblastoma mouse models, where tumor control with prolonged survival of mice with GL261/HER2 tumors was achieved [27]. Based on these results, we are currently performing the phase I, first-in-human clinical trial CAR2BRAIN (NCT03383978, clinicaltrials.gov) to explore an adoptive cellular immunotherapy with local application of NK-92/5.28.z cells in patients suffering from a relapse of a HER2-positive glioblastoma [22].

Based on of the considerations discussed above, we here investigated combination therapy with local CAR-NK cell treatment using clonal NK-92/5.28.z cells and systemic anti-PD-1 checkpoint inhibition in advanced GL261/HER2 tumors. We explored the effect of the combination immunotherapy on survival and analyzed the modulation of the tumor microenvironment.

## Material and methods

### Flow cytometry

Expression of extracellular and intracellular proteins of tumor cells and splenocytes was determined using fluorescence-coupled antibodies [30]. A full list of antibodies and panels is provided in the supplementary [Supplementary Table 1]. Briefly, cells were harvested and washed with FACS buffer (PBS + 2% FCS) and incubated with the corresponding antibodies for 45 min at 4 °C. For intracellular staining cells were fixed and permeabilized prior to staining. Cells were analyzed after two further washing steps with FACS buffer. Cell viability was analyzed with propidium iodide (PI) as previously described [31]. For flow cytometry analysis, a FACSCanto II flow cytometer (BD Biosciences, Heidelberg, Germany) or a FACSymphony A5SE flow cytometer (BD Biosciences, Heidelberg, Germany) were used.

### Murine glioblastoma models

All in vivo experiments were carried out in accordance with the guidelines and regulations of the German animal protection law upon approval by the responsible government committee (Regierungspräsidium Darmstadt, Darmstadt, Germany, approval number FK-1088). For subcutaneous tumor models 1 × 10^6^ syngeneic GL261/HER2 murine glioma cells were injected into the right flank. For orthotopic glioblastoma models, anesthetized mice were immobilized in a stereotaxic fixation device (Stoelting, Wood Dale, IL, USA) and injected with 1 × 10^5^ GL261/HER2 tumor cells in 2 μL PBS in the right hemisphere.

Subcutaneous tumors were treated by intratumoral injection of 1 × 10^7^ NK-92/5.28.z cells in 100 µL. Orthotopic glioblastoma tumors were treated by stereotactic injection of 2 × 10^6^ NK-92/5.28.z cells in 3 µL as described previously [32]. For both, intracranial and subcutaneous tumor models, 250 µg anti-PD-1 antibody or 250 µg IgG Isotype (Bio X Cell, Lebanon, NH, USA) were injected intraperitoneally in 50 µL injection buffer (supplied by Bio X Cell, Lebanon, NH, USA).

For subcutaneous experiments mice received their first treatment after reaching the inclusion criteria of a minimal tumor volume of 40 mm^3^. Figures show time expired from start of therapy onwards. NK-92/5.28.z cells were injected locally into the tumor at day 0 (inclusion date), 7 and 14, and anti-PD-1 antibody was injected intraperitoneally (i.p.) on days 0, 3, 7, 10, 14 and 17. For mice investigating the abscopal effect the same treatment scheme was applied after both tumors (primary and contralateral) reached the inclusion criteria of a minimal tumor volume of each 40 mm^3^.

For the advanced orthotopic intracranial glioma mouse model, tumor cells were allowed to engraft for 8 days. MRI was performed at day 7 and only mice with detectable tumors were used for the experiments. NK-92/5.28.z cells were injected locally via the burr hole at days 8, 15 and 22, and anti-PD-1 antibody was injected intraperitoneally (i.p.) on days 8, 12, 15, 19, 22 and 26.

### Multispectral imaging

Multispectral stainings were performed on sections of FFPE-fixed tumor tissue with respective antibodies using an automated staining instrument. For image acquisition Vectra Polaris instrument (Akoya Biosciences Inc.) was used. Image analysis was performed via HALO™ software (Indica Labs, Albuquerque, NM).

### Statistical analysis

Quantitative data are expressed as mean and standard of the mean (SEM), if not otherwise stated. Statistical significance was determined with a one-tailed Student’s *t* test. If multiple sample groups were compared, significance was determined by ANOVA test. *p* < 0.05 was regarded as significant. All analyses were performed using GraphPad Prism (Version 10.1.0; GraphPad Software, La Jolla, CA, USA).

## Results

### NK-92/5.28.z cell-mediated lysis of HER2-positive glioblastoma cells induces PD-L1 expression through IFNγ secretion

**Fig. 1:**
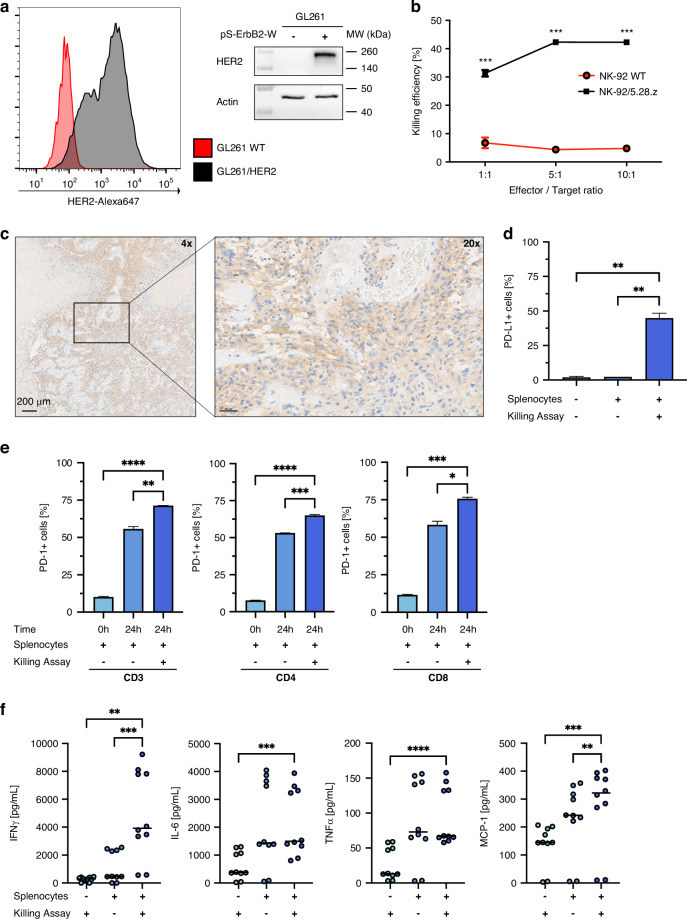
NK-92/5.28.z cell mediated lysis of HER2-positive GB cells. **a** Expression of human HER2 in the GL261 glioma cell line; (left) flow cytometric analysis, (right) Western blot. **b** Specific lysis of NK-92/5.28.z cells compared to NK-92 wildtype (WT) cells targeted against GL261/HER2 cells (*n* = 3; one-tailed Student’s *t* test. ***p < 0.001). **c** IHC staining for PD-L1 in glioblastoma relapse tissue from a patient participating in the CAR2BRAIN trial. **d** In vitro induction of PD-L1 on GL261/HER2 cells upon stimulation in a co-culture assay measured via flow cytometry (*n* = 3, mean ± SEM; ANOVA with Sidák’s multiple comparison test. **p < 0.01). **e** Induction of PD-1 expression on ex vivo isolated CD3, CD4 and CD8 T cells upon stimulation in a co-culture assay measured via flow cytometry (*n* = 3, mean ± SEM; ANOVA with Sidák’s multiple comparison test. *p < 0.05, **p < 0.01, ***p < 0.001, ****p-value < 0.0001). **f** In vitro secretion of pro-inflammatory cytokines of isolated murine splenocytes upon stimulation in a co-culture assay measured via flow cytometry (*n* = 10, scatter dot blot and median expression; ANOVA with Sidák’s multiple comparison test. *p < 0.05, **p < 0.01, ***p < 0.001, ****p < 0.0001).

Glioblastoma cells frequently express HER2, rendering this antigen an attractive target for a CAR-directed immunotherapeutic approach. Murine GL261 glioma cells were transduced with a lentiviral vector encoding human *HER2* to enable further testing of the NK-92/5.28.z cell product currently under clinical investigation in the CAR2BRAIN trial in in vivo experiments in the syngeneic C57BL/6 mouse model [22, 32] [Fig. 1a].

In vitro cell killing assays demonstrated high specific cytotoxic activity of NK-92/5.28.z against GL261/HER2 cells, while HER2-negative GL261 control cells were not lysed [Fig. 1b]. Parental NK-92 cells showed only little basal cytotoxicity towards both, GL261/HER2 and GL261 control cells [data not shown].

To demonstrate the activation of the PD-1/PD-L1 system in glioblastoma, IHC staining of tumor tissues from patients of the clinical trial CAR2BRAIN was performed, where robust PD-L1 upregulation was found already prior to study treatment [Fig. 1c].

To investigate the effects of NK-92/5.28.z cell therapy on the PD-1/PD-L1 system, in vitro co-culture assays were performed, and expression of human HER2, and murine PD-1 (mPD-1) and PD-L1 (mPD-L1) was analyzed by flow cytometry. GL261/HER2 cells were co-incubated with murine splenocytes in the bottom well. In the inserted chamber, GL261/HER2 cells were co-incubated either with or without NK-92/5.28.z cells to induce killing as well as cytokine and antigen release [Supplementary Fig. 1A]. We only found low expression of mPD-1 on CD3^+^ splenocytes and mPD-L1 on HER2-positive GL261/HER2 cells in the absence of NK-92/5.28.z cells in the chamber. In contrast, killing of GL261/HER2 cells by NK-92/5.28.z cells in the insert increased expression of mPD-1 on CD3^+^ splenocytes and mPD-L1 on GL261/HER2 cells co-incubated in the bottom of the wells [Fig. 1d, e].

We measured the release of several pro- and anti-inflammatory murine cytokines by the respective splenocytes in co-culture assays to elucidate mediators of increased mPD-1 and mPD-L1 expression. We found a significant increase of murine IFNγ (mIFNγ) and murine MCP-1 (mMCP-1) secretion after co-incubation of NK-92/5.28.z cells with tumor cells in the insert. Furthermore, there was no increase in concentrations of the pro-inflammatory cytokines murine IL-6 (mIL-6) and murine TNFα (mTNFα) [Fig. 1f]. These results show that the interaction of NK-92/5.28.z cells and glioma cells trigger an adaptive/evasive upregulation of the PD-1/PD-L1 system mediated at least in part by mIFNγ. This is supported by the strong induction of mPD-L1 by direct co-incubation with mIFNγ in vitro [Supplementary Fig. 1B]. Therefore, PD-L1 induction represents a mechanism of adaptive resistance induced after monotherapy with CAR-NK cells.

### Efficacy of combination therapy with NK-92/5.28.z cells and anti-PD-1 antibody in the syngeneic subcutaneous GL261/HER2 tumor model

**Fig. 2:**
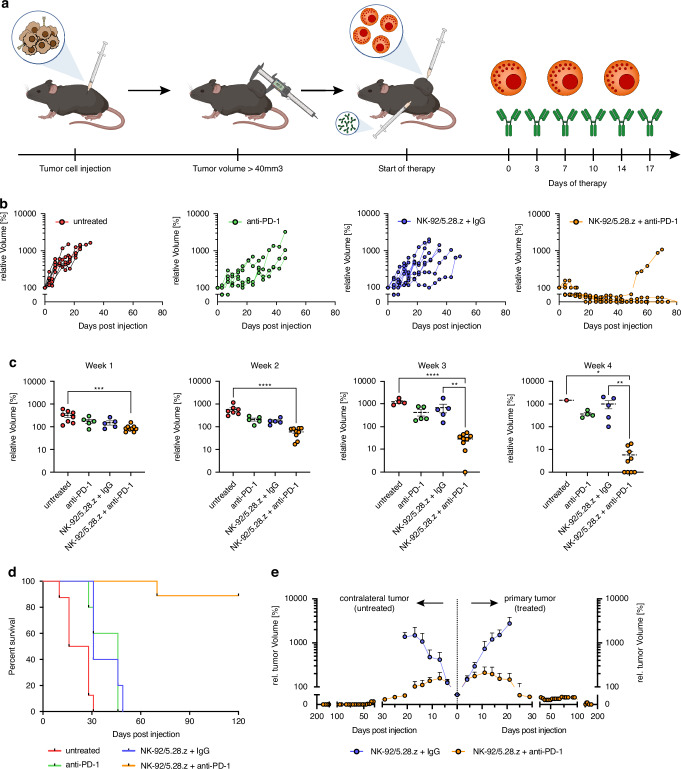
Efficacy of the combination of local NK-92/5.28.z therapy with systemic anti-PD-1 checkpoint inhibition on subcutaneous GL261/HER2 tumors. **a** Schematic of the treatment regimen for tumor-bearing mice. **b** Spider plot of relative tumor growth of all different treatment groups. **c** Growth comparison of different treatment groups between week 1 and week 4 (left to right) (each point represents one mouse; scatter dot plot with mean volume ±SEM; ANOVA with Tukey’s multiple comparison test. *p < 0.05, **p < 0.01, ***p < 0.001, ****p < 0.0001). **d** Kaplan–Meier survival analysis of all treatment groups (*n* = 5–10, Log-Rank (Mantel–Cox) test. ****p < 0.0001). **e** Relative tumor growth of primary and contralateral tumors demonstrate the induction of an “abscopal effect”. Only the primary tumor was locally treated with NK-92/5.28.z cells; all mice received additional IgG or anti-PD-1 systemically (*n* = 3, mean ± SEM).

To investigate whether disruption of the PD-1/PD-L1 axis can enhance the efficacy of NK-92/5.28.z cell therapy in vivo, combination therapy of NK-92/5.28.z with systemic anti-PD-1 checkpoint inhibition was first evaluated in the subcutaneous syngeneic GL261/HER2 model in C57BL/6 mice. After initial tumor cell injection, tumor size was regularly measured using a Caliper. When mice met their inclusion criteria of minimal tumor volume, they were treated following the therapy scheme shown in Fig. 2a.

We tested therapy conditions with advanced-stage tumors, for which NK-92/5.28.z monotherapy was not effective [Fig. 2b]. While parental NK-92 cells and anti-PD-1 antibody alone as well as NK-92/5.28.z cells with isotype-matched control antibody and the combination of parental NK-92 cells with anti-PD-1 checkpoint inhibition all had no or little effect, the combination of NK-92/5.28.z cells and checkpoint inhibition was highly efficacious and resulted in tumor rejections with long-term remissions in the majority of treated animals. Interestingly, a delay in tumor growth and ultimately tumor regressions became apparent only after completion of the therapy regimen, suggesting an indirect mechanism of action mediated by the induction of an endogenous immune response [Fig. 2b, c and Supplementary Fig. 2A].

Kaplan–Meyer analysis of symptom-free survival demonstrated strongly increased activity with a synergistic effect of the combination of local therapy with NK-92/5.28.z cells and systemic anti-PD-1 checkpoint blockade as compared to NK-92/5.28.z monotherapy, anti-PD-1 monotherapy, and the untreated control group (***p < 0.001) [Fig. 2d].

Long-term survival after CAR-NK cell treatment resulted in a robust long-term immune memory as already shown in rechallenge experiments performed by Zhang and Burger et al. [27]. Since long-term memory has already been characterized, we here focused on the immunotherapeutic effect induced. Therefore, we investigated the emergence of an “abscopal effect”, i.e., a therapeutic effect observed in distant tumor locations not directly treated with CAR-NK cells. Hence, GL261/HER2 tumors were implanted subcutaneously in both flanks of C57BL/6 mice. Only one tumor was treated either locally with NK-92/5.28.z cells alone or in combination with systemic anti-PD-1 antibody. Then the effect on the contralateral not locally treated tumor was assessed. In line with our previous results, the combination of NK-92/5.28.z and anti-PD-1 was able to induce tumor regression of the locally treated tumor. Moreover, combination therapy resulted in regression also of the contralateral tumor, ultimately leading to long-term survival [Fig. 2e and Supplementary Fig. 2B–D].

### Efficacy of combination of local therapy with NK-92/5.28.z cells and systemic anti-PD-1 checkpoint blockade in orthotopic tumor grafts in syngeneic mice

**Fig. 3:**
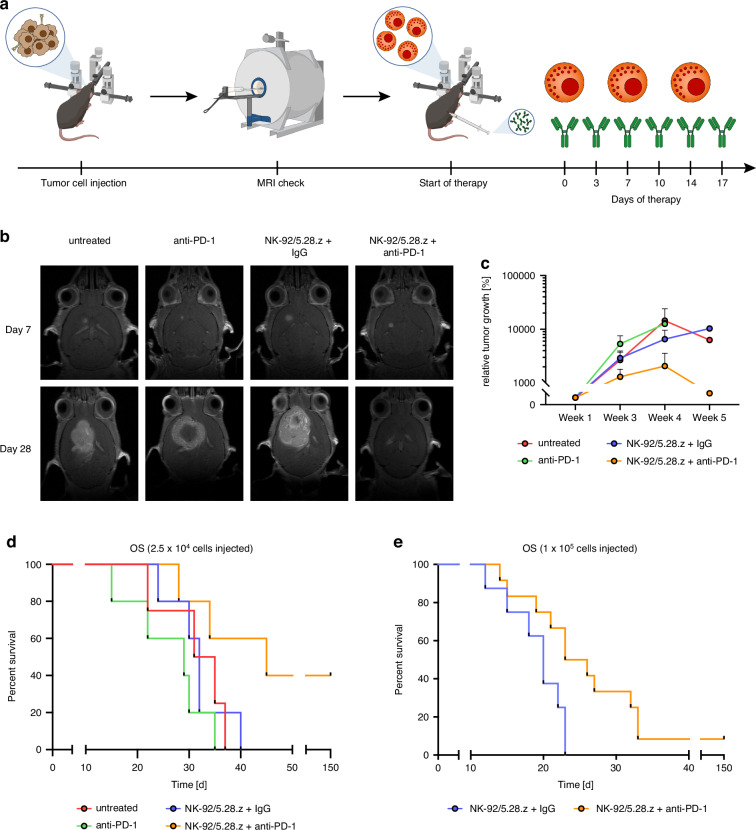
Efficacy of combination therapy with NK-92/5.28.z cells and anti-PD-1 antibody in orthotopic tumors. **a** Schematic of the treatment regimen for tumor-bearing mice. **b** Representative MRI images taken on days 7 and 28 after tumor cell injection. **c** Relative tumor growth over time measured via MRI. **d** Kaplan–Meier survival analysis of all treatment cohorts after therapy of advanced tumors (*n* = 5, Log-Rank (Mantel–Cox) test, *p* = 0.0640). **e** Kaplan–Meier survival analysis of combination therapy of NK-92/5.28.z and anti-PD-1 compared to NK-92/5.28.z with control IgG after treatment of large late-stage tumors (*n* = 8–12, Log-Rank (Mantel–Cox) test, *p* = 0.0476).

To evaluate the efficacy of the combination therapy with NK-92/5.28.z and systemic anti-PD-1 checkpoint blockade in an orthotopic intracranial model, 2.5 × 10^4^ GL261/HER2 cells were injected into the right striatum of syngeneic C57Bl/6 mice, and treatment was performed as shown in Fig. 3a. After verification of successful engraftment via MRI, treatment was initiated at day 8 after tumor cell injection. Treatment with anti-PD-1 antibody alone or NK-92/5.28.z cells with isotype-matched control antibody did not delay growth in these advanced tumors as analyzed by serial MRI measurements [Fig. 3b] and did not prolong symptom-free survival as compared to untreated mice. However, the combination of NK-92/5.28.z cells and anti-PD-1 antibody was highly efficacious, resulting in a significantly reduced tumor size [Fig. 3c] and prolonged survival compared to untreated mice or mice treated with monotherapy [Fig. 3d]. After successful treatment of advanced-sized tumors, this approach was evaluated for even larger-sized tumors. Injection with an increased number of tumor cells (1 × 10^5^ cells) was followed by therapy initiation at day 8 after verification of tumor engraftment by MRI. NK-92/5.28.z cells with anti-PD-1 antibody were compared to NK-92/5.28.z with control IgG to focus on the effects of the combination therapy. Again, even under these more challenging conditions, the combination therapy resulted in significantly prolonged survival (*p* = 0.0476) [Fig. 3e].

### Modification of the tumor immune microenvironment induced by combination treatment with NK-92/5.28.z cells and anti-PD-1 checkpoint inhibition

**Fig. 4:**
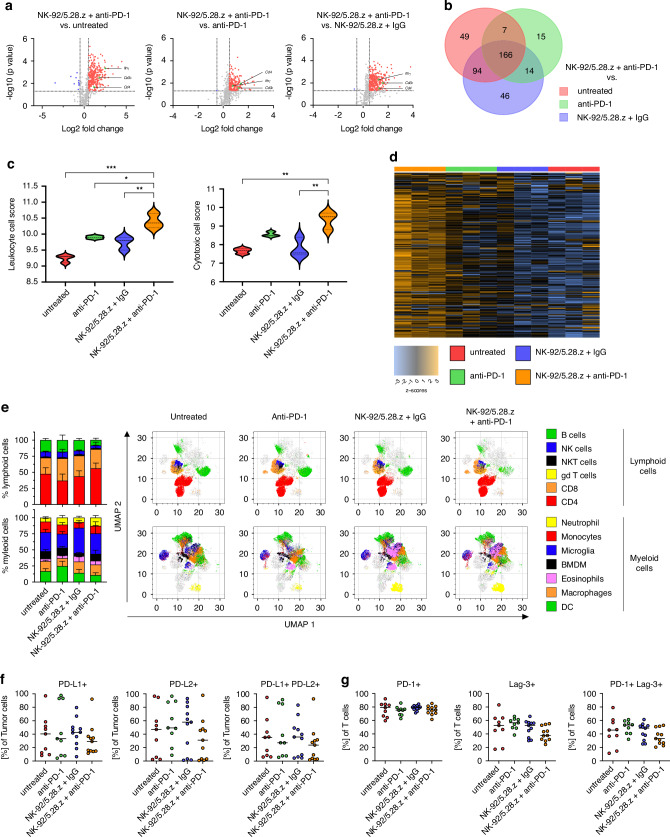
Modification of the tumor immune microenvironment induced by combined NK-92/5.28.z cells and anti-PD-1 antibody treatment. **a**–**d** Analyses of FFPE samples of orthotopic tumors using NanoString RNAseq data with comparison of differentially expressed genes after NK-92/5.28.z and anti-PD-1 combination therapy with all other treatment regimens in (**a**) Volcano plot and (**b**) Venn diagram. **c** Calculated cytotoxic and leukocyte cell scores using NanoString software. **d** Heatmap of differentially expressed genes associated with T cell pathways. **e**–**g** Flow cytometric analysis of explanted orthotopic tumors. **e** Cell counts of lymphoid and myeloid cell infiltration after treatment. **f** PD-L1-, PD-L2- and double-positive tumor cells after therapy. **g** PD-1-, Lag-3- and double-positive T cells after therapy.

Regression of subcutaneous tumors occurred only towards the end of the therapy regimen in mice treated with NK-92/5.28.z cells and anti-PD-1 checkpoint blockade, suggestive of for a mechanism involving an endogenous immune response commenced indirectly by the combination therapy. Therefore, focusing on the more relevant orthotopic tumors, we analyzed the tumor microenvironment (TME) for potential changes induced by the treatment. Hence, we applied NanoString RNAseq to evaluate the cellular immune composition of the tumor microenvironment. Analysis using the via NanoString Pan Cancer Immune Panel showed that NK-92/5.28.z cell therapy and anti-PD-1 treatment led to a high number of differentially expressed genes compared to all control groups [Fig. 4a]. In total 166 genes were differentially expressed in all comparisons, i.e., NK-92/5.28.z and anti-PD-1 against untreated, anti-PD-1 and NK-92/5.28.z and isotype IgG control cohorts [Fig. 4b], with the most prominent differences in cytotoxicity-related genes, e.g., *Ifnγ*, *Cd3ε* and *Cd4*. Moreover, all therapy cohorts displayed defined clustering in a principal component analysis, with combination therapy samples clearly separated from all other treatment groups [Supplementary Fig. 3A].

NanoString RNAseq measurements allowed us to further analyze immune-related pathways. Both NK-92/5.28.z cells and control antibody or monotherapy with anti-PD-1 checkpoint inhibition led to some minor differences in immune composition when compared to untreated mice. However, the combination of NK-92/5.28.z cells and anti-PD-1 resulted in the most prominent effects, with increased CD45 leukocyte and cytotoxic cell score [Fig. 4c].

Analysis of immune-related pathways demonstrated that the combination therapy of NK-92/5.28.z cells and anti-PD-1 checkpoint inhibition induced a strong upregulation of pro-inflammatory pathways. Notably, pro-inflammatory functions of “T cells” and “*Ifnγ*”-related pathway genes were increased. Consistent with overall clustering in PCA plots, samples obtained from the combination therapy group clustered together and were clearly separated from monotherapy and untreated cohorts [Fig. 4d and Supplementary Fig. 3B, C].

After bulk RNAseq, a more quantitative approach via flow cytometry was deployed to facilitate quantification of the cellular composition. The gating strategy is shown in Supplementary Fig. 4. As depicted in Fig. 4d, high immune cell infiltration into the tumor was observed and all major immune cell populations were detected. Clustering and gating analysis of dimensionally reduced data showed a clear separation of respective immune cell populations within the tumor of all treatment groups. Furthermore, differences in immune cell composition between the different treatment cohorts were detected [Fig. 4e]. Both monotherapy and combination therapy resulted in increased levels of CD45^+^ leukocytes compared to untreated mice [Supplementary Fig. 5A]. Interestingly, all treatment regimens also led to a reduced number of CD45^-^ stromal cells within the TME, hence resulting in an increased CD45 ratio of infiltrating cells [Supplementary Fig. 5B, C].

Upregulation of the checkpoint protein PD-L1 is frequently found in GB [Fig. 1c]. The surrounding tumor microenvironment typically demonstrates a strong increase of the exhaustion markers PD-1 and Lag-3 [33, 34]. Therefore, we explored the expression of the checkpoint molecules PD-1 and PD-L1, among other factors, with flow cytometry. Thereby, mPD-L1 and murine PD-L2 (mPD-L2) expression on HER2-positive tumor cells was highly prevalent [Fig. 4f]. Correspondingly, infiltrating T cells demonstrated high mPD-1 and murine LAG-3 (mLAG-3) expression in all treatment cohorts, albeit with a trend towards a lower mLAG-3 expression in the NK-92/5.28.z and anti-PD-1 combination therapy cohort [Fig. 4g].

### Superior treatment effects of NK-92/5.28.z cells and anti-PD-1 checkpoint inhibition are characterized via a CD4^+^ T cell immune response

**Fig. 5:**
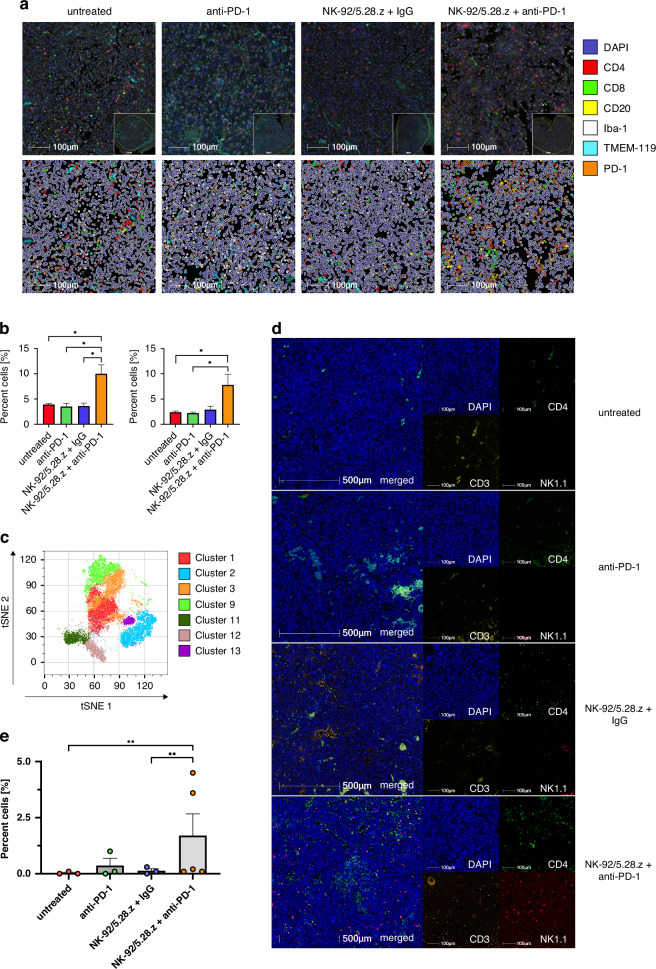
Combination therapy with NK-92/5.28.z and anti-PD-1 checkpoint inhibition induces a CD4-driven T-cell response. **a** Representative multiplex IHC images of untreated, anti-PD-1, NK-92/5.28.z + IgG and NK-92/5.28.z + anti-PD-1 treated mice (left to right). Proteins stained on FFPE slides are indicated on the right. Upper panels depict IHC images, lower panels after applying an analysis grid for better visualization of cell types. **b** Quantification of CD4^+^ plus CD8^+^ cells (left) and CD4^+^ cells (right) using the PhenOptics data (*n* = 3, one-tailed Student’s *t* test. * *p* < 0.05). **c** Cluster analysis of intracellular flow cytometry data; the t-SNE dimensionally reduced plot shows different clusters based on the Phenograph algorithm. **d** Representative multiplex IHC images of untreated, anti-PD-1, NK-92/5.28.z + IgG and NK-92/5.28.z + anti-PD-1 treated mice. Slides were stained for CD3, CD4 and NK1.1, respectively, with merged images and single stainings shown. **e** Quantification of CD4 and NK1.1 double-positive cells from the multiplex images shown in (**d**) (*n* = 3–5, o*n*e-tailed Student’s *t* test. ** *p* < 0.01).

Further analysis of the TME via multiplex IHC staining of treated GL261/HER2 tumors further confirmed significant differences between the treatment cohorts, especially regarding the infiltration of T cells. Monotherapy with anti-PD-1 or NK-92/5.28.z cells combined with control antibody did not increase T cell infiltration compared to untreated mice. In contrast, combination therapy with NK-92/5.28.z cells and anti-PD-1 considerably increased intratumoral T cell infiltration. This was mainly due to a strong increase in the number of CD4^+^ T cells [Fig. 5a, b], with an only modest increase of CD8^+^ T cells [data not shown].

Thus, we explored the induction of pro-inflammatory T cell subtypes by the combination treatment. For this, T cell subpopulations isolated from treated GL261/HER2 tumors were analyzed by intracellular flow cytometry to further specify the immune response induced. We detected intratumoral infiltration of all CD4^+^ and CD8^+^ T cell subtypes, including T helper 1 (Th1), Th2, Th9, Th17, Th22 and regulatory T cells [Supplementary Fig. 6A]. Applying the Phenograph algorithm [35] to the intracellular flow cytometry data, we identified 15 distinct clusters. Further analysis revealed seven clusters which showed divergent cell counts between the treatment groups and untreated mice [Fig. 5c and Supplementary Table 2]. Notably, clusters of interest differed by their CD4 and CD8 expression. Three cluster populations expressed high levels of CD4, while two populations strongly expressed CD8, respectively. Interestingly, one cluster of T cells was double positive, and another cluster was double negative for CD4 and CD8.

Out of these seven clusters, three more relevant clusters of interest were identified. Untreated control mice showed a significant enrichment in cluster C12, with a phenotype of regulatory CD8^+^ T cells, characterized by high expression of murine CD8, murine CD25 and murine FOXP3 [36], as well as memory CD8^+^ T cells, characterized by high expression of murine CXCR5. Interestingly, these cells also showed the highest PD-1 and IFNγ expression [Supplementary Fig. 6B]. Cells in clusters C1 and C3, however, were most abundant in mice treated with the combination of NK-92/5.28.z cells and anti-PD-1 checkpoint inhibition. These two clusters were mainly composed of CD4^+^ T cells, with cluster C3 representing naïve CD4^+^ T cells. In contrast to C3, based on the expression profile of cytokines and membrane proteins, cluster C1 appeared to be composed of CD4^+^ NKT cells. To evaluate this further, we used multiplex IHC staining of CD3, CD4 and NK1.1 [Fig. 5d]. In mice treated with the combination of NK-92/5.28.z cells and anti-PD-1 checkpoint inhibition, a significant increase in CD4^+^ cells were detected, both at pre-defined time points, and in endpoint analyses. Supporting our hypothesis, the same effect was observed for CD3^+^ and NK1.1^+^ cells [Supplementary Fig. 6C]. Whereas hardly any CD4^+^NK1.1^+^ double-positive cells were present in untreated mice or mice treated with NK-92/5.28.z and control IgG, a small increase was detected in anti-PD-1 treated mice. However, only mice treated with NK-92/5.28.z in combination with anti-PD-1 checkpoint blockade displayed a strong increase in infiltrating CD4^+^NK1.1^+^ cells [Fig. 5e]. To explore if similar effects also occur in patients suffering from glioblastoma, we analyzed tumor tissue from the phase I clinical CAR2BRAIN trial. Immunohistology of biopsies directly prior to the start of a combination therapy with NK-92/5.28.z and anti-PD-1 checkpoint blockade showed low levels of infiltrating CD3^+^CD4^+^CD161^+^ NKT cells. After initial biopsy, patients received repetitive injections of CAR-NK cells into the resection cavity via a Rickham catheter combined with intravenous anti-PD-1 checkpoint inhibition with ezabenlimab. Tumor relapse surgery was scheduled after four weeks. In post-treatment tumor tissue, we detected a considerable increase of NKT cells compared to tissue sampled directly prior to therapy initiation in both patients where tissue pairs were evaluable [Supplementary Fig. 7]. The comparable effects from CAR-NK cell therapy combined with checkpoint inhibition both in murine tumor tissue and in patient material substantiate the increase of NKT cells as a specific effect of this therapeutic approach with clinical relevance [22].

## Discussion

Glioblastoma is a tumor characterized as rather immunologically “cold”, with a low immunogenicity of tumor cells and a pronounced immunosuppressive tumor immune microenvironment (TME) [37, 38]. To achieve a therapeutic effect, immunotherapeutic approaches would have to surmount these obstacles. An increased release of tumor antigens as well as cytokines induced by CAR-mediated tumor cell lysis might trigger an immune reaction, which may be unblocked by immune-checkpoint inhibition in a combination therapy approach.

Here, we demonstrate the synergistic activity of NK-92/5.28.z CAR-NK cells in combination with anti-PD-1 checkpoint inhibition for treating advanced-stage HER2-positive glioblastoma. We verified that NK-92/5.28.z potently lyse glioma cells expressing human HER2 in vitro as previously demonstrated [32]. Monotherapy with NK-92/5.28.z cells induced rejection of smaller GL261/HER2 tumors and thereby conveyed lasting immunity against GL261 rechallenge in long-term surviving mice [27]. Previous work suggests that adoptive transfer of NK-92/5.28.z in vivo triggers the release of tumor antigens with subsequent activation of immune cells, most likely augmented by the secretion of high levels of pro-inflammatory cytokines, and ultimately induces a long-lasting anti-tumor response [27, 39]. Here, we observed an increased release of pro-inflammatory and potentially anti-tumorigenic cytokines by murine immune cells induced upon CAR-NK cell-mediated tumor cell lysis. Thereby, the production of pro-inflammatory cytokines like IFNγ is particularly important in the context of effector T cell activation and inhibition of regulatory T cells [40, 41]. Moreover, the lack of IL-6 secretion, which potentially mediates *cytokine release syndrome* (CRS) and *immune effector cell associated neurotoxicity syndrome* (ICANS), suggests that CAR-NK cells may be better tolerated as compared to CAR-T cells. However, local release of pro-inflammatory IFNγ also increases PD-L1 expression on tumor cells [4, 32, 42]. GL261 normally expressing low levels of PD-L1 in vitro, strongly upregulated PD-L1 expression mediated by IFNγ stimulation mediated by splenocytes. In vivo the expression of PD-L1 and PD-L2 was per se considerably higher, but with only minor differences between treatment groups. This effect might be explained by continuous IFNγ stimulation of tumor cells in vivo even in the absence of CAR-NK cells. Further, sustained antigen presentation to T cells leads to increased PD-1 levels [4]. The upregulation of checkpoint proteins can contribute to the distinctive immunosuppression within the TME [43–45] and is an established mechanism in glioblastoma [46]. This was also detected in tissue samples from the ongoing clinical trial CAR2BRAIN exploring NK-92/5.28.z cell therapy in glioblastoma relapse patients. Growing tumors with advanced size over time almost inevitably become less susceptible to NK-92/5.28.z cell therapy due to the increasingly unfavorable numerical ratio between tumor and effector cells as well as incremental intratumoral immunosuppression. Accordingly, the efficacy of NK-92/5.28.z cell therapy against late-stage tumors might be enhanced by targeting immunosuppressive mechanisms within the tumors, providing a rationale for respective combination therapy.

We therefore evaluated the effect of adoptive local CAR-NK cell therapy in combination with systemic anti-PD-1 checkpoint inhibition. In the immunocompetent mouse glioma model with advanced GL261 tumors, CAR-NK therapy, and anti-PD-1 checkpoint inhibition alone were not sufficient to induce robust anti-tumor responses, likely due to the pronounced immunosuppressive TME in advanced tumors [47]. Indeed, only the combination of both treatments was able to induce pro-inflammatory alterations in the TME in larger tumors. This resulted in complete tumor rejection and long-term survival of mice bearing subcutaneous tumors. Interestingly, we observed that tumor growth was not instantly reversed by the combination therapy. Instead, tumor expansion was halted, and regression occurred only after treatment completion, suggesting therapy-mediated induction of an endogenous anti-tumor immune response, as previously shown for smaller tumors [48]. We validated these results in an orthotopic intracranial GL261/HER2 glioma model, which mimics the typical immunosuppressive TME of human glioblastomas more closely. Correspondingly, efficient anti-tumor responses were achieved combining local injection of CAR-NK cells with systemic checkpoint inhibition. Allowing a further increase in the tumor volume before treatment initiation limited the effect of the combination therapy, while nevertheless we still achieved complete tumor regression in some of the animals.

Previous experiments with smaller tumors have already shown that long-term surviving mice cured by CAR-NK cell therapy had acquired robust protection against tumor rechallenge, which was reverted by T cell depletion [48]. Thereby, NK-92/5.28.z therapy had induced a broad endogenous immune reaction with both an antibody and T cell response, targeted against antigens unrelated to HER2 as indicated by rejection of GL261/HER2 as well as HER2-negative parental GL261 cells. Our analysis of orthotopic tumors by NanoString RNAseq confirmed an accentuated pro-inflammatory immune response of mice treated with NK-92/5.28.z monotherapy compared to untreated mice. Moreover, our findings show that this endogenous immune response can be even further enhanced when combining CAR-NK cell therapy with anti-PD-1 checkpoint inhibition. This clearly demonstrates the capacity of the combination therapy to overcome the immunosuppressive TME.

Bulk RNAseq via Nanostring allowed us to analyze intratumoral immune signaling. Pathway analysis demonstrated an increased pro-inflammatory immune cell phenotype after combination therapy. We explored if this was also accompanied by an increase in intratumoral abundancy of CD8^+^ T cells. Interestingly, while the combination therapy increased the number of CD4^+^ T cells, this was not the case for CD8^+^ T cells. This result is consistent with previous preclinical work that demonstrated a crucial role of the CD4^+^ T cells in the response of GB to checkpoint inhibition [49]. Aslan et al. [47]. investigated mice responding or not responding to immune checkpoint inhibition. Congruent with our current results, the therapeutic effect of anti-PD-1 and anti-CTLA4 was conditional on a CD4-driven anti-tumor response, while depletion of CD4^+^ cells still had a less pronounced effect on overall survival than depletion of CD8^+^ cells. Also, responding mice were characterized by fewer infiltrating myeloid cells, and the therapeutic effect of checkpoint inhibition depended on the PD-L1/CD80 interaction of myeloid cells and CD4^+^ T cells [47]. To explore the possible contribution of CD4^+^ T cells to the anti-tumor effect in our setting, infiltrating CD4^+^ T cells were analyzed by intracellular cytokine staining and flow cytometry to distinguish their individual subpopulations. CD4^+^ T cells are generally classified into different subsets, e.g., T helper cell 1 (Th1), Th2 and regulatory T cells (Treg) [50], and the ratio of Th1/Th2 cells is an established determinant for GB prognosis [51, 52].

Unsupervised clustering was used to explore the flow cytometry data [35]. Thereby, NK-92/5.28.z and checkpoint inhibitor monotherapies as well as the combination therapy showed reduced levels of regulatory CD8^+^ T cells. The most abundant cluster in Phenograph analysis revealed an expression profile characteristic for CD4^+^ NKT cells. NKT cells are CD4^+^ T cells co-expressing NK-cell specific markers like NK1.1 or its human homolog CD161. In contrast to T cells, NKT cells are CD1d restricted [53, 54]. CD1d is a class Ib MHC-like protein, which presents lipid antigens instead of peptide epitopes. Lipids presented by CD1d are recognized by a specific type of *αβ* T-cell receptors (TCR*αβ*) with very limited variety. Generally, NKT cells can be divided into type I and II NKT cells. Type I NKT cells, also known as invariant NKT cells (iNKT), express a specific TCR*α* chain (gene segments V*α*14J*α*18) and only few different TCR*β* chains (variable gene segments V*β*2, 7 and 8). Unlike conventional T cells which are restricted to specific peptide antigen structures, NKT cells can recognize a broad variety of lipid antigen structures. Typically, type I NKT cells recognize glycosphingolipids or phospholipids (e.g., *α*-galactosylceramide (*α*-GalCer)) [53]. The role of iNKT cells is linked to an increased anti-tumor immune response through production of IFN*γ* or IL-12. This is mediated via direct killing through NKG2D and the CD1d-TCR interaction. Further, CD1d-TCR interaction can induce DC activation, M1 polarization of macrophages and terminal differentiation of MDSCs [53]. In contrast, type II NKT cells rather mediate immunomodulatory and anti-inflammatory responses of other immune cells through secretion of several anti-inflammatory cytokines [55]. According to our data, it appears likely that type I NKT cells are attracted to the tumor site in mice treated with NK-92/5.28.z in combination with anti-PD-1 checkpoint blockade. Thereby, we detected highly increased levels of CD4^+^NK1.1^+^ double-positive cells only in mice treated with combination therapy, while levels of infiltrating CD4^+^NK1.1^+^ cells were comparably low in animals of the monotherapy and the control cohorts.

Even though GL261 cells are known to be rather immunogenic due to their initial generation via chemical induction [56, 57], only the combination therapy led to sufficient immune cell activation in the TME to prolong survival, while responses triggered by anti-PD-1 and CAR-NK monotherapies alone were not sufficient to overcome the immunosuppressive TME of advanced tumors. For our experiments, we selected a therapeutic intervention with human NK-92/5.28.z CAR-NK cells targeting murine GL261 glioma cells that carry human *HER2*, which poses an interspecies barrier. This limitation of our model can affect both, the cytokines released and ligand-receptor interactions, which may have influenced the observed changes in the TME and the response to treatment. Nevertheless, while in the chosen setting monotherapy with NK-92/5.28.z cells did not induce a meaningful alteration of the immunosuppressive TME, combination therapy with anti-PD-1 resulted in considerable changes in the tumor immune cell infiltrate and markedly extended survival. Noteworthy, these data obtained with the same NK-92/5.28.z cell clone that is currently assessed in our ongoing phase I clinical trial CAR2BRAIN facilitates rapid clinical translation of the combination therapy approach.

In summary, our data clearly demonstrate that in contrast to respective monotherapies, HER2-targeted NK-92/5.28.z CAR-NK cells applied in combination with anti-PD-1 checkpoint inhibition efficiently induces tumor regression of advanced tumors in the immunocompetent GL261/HER2 mouse model, accompanied by alterations in the glioma-specific tumor microenvironment consistent with a switch from an immunologically “cold” to an immunologically more accessible tumor. This modulation of GB TME induced only by the combination therapy, but not the monotherapies, is a pivotal condition for successful immunotherapeutic intervention.

## Supplementary information

Supplementary Material

## Data Availability

Data supporting the findings of this manuscript are available within this manuscript and the supplementary material.
