# Supplementary material for: CAR-NK cell therapy combined with checkpoint inhibition induces an NKT cell response in glioblastoma
Source: Br J Cancer. 2025 Mar 18;132(9):849–60. doi: 10.1038/s41416-025-02977-8 (PMC12041480; doi:10.1038/s41416-025-02977-8)
Supplement: Supplementary file 1 — Supplementary Material [file 41416_2025_2977_MOESM1_ESM.docx]

**Supplementary Material**

**CAR-NK cell therapy combined with checkpoint inhibition induces an NKT cell response in glioblastoma**

Strassheimer F, Elleringmann P, Ludmirski G, Roller B, Macas J, Alekseeva T, Cakmak P, Aliraj B, Krenzlin H, Demes MC, Mildenberger IC, Tonn T, Weber KJ, Reiss Y, Plate KH, Weigert A, Wels WS, Steinbach JP, and Burger MC

**Supplementary Materials and Methods**

**Cell culture**

Established murine GL261 glioma cells were cultured in Dulbecco´s modified Eagle´s medium (DMEM, Gibco, Darmstadt, Germany) containing 10% fetal calf serum (FCS; Biochrom KG, Berlin, Germany), 100 IU/ml penicillin and 100 µg/ml streptomycin (Life Technologies, Karlsruhe, Germany) in cell culture incubators at 37 °C and 5% CO_2_. GL261/HER2 cells were generated via lentiviral transduction of GL261 with HER2 as previously described.^1^ NK-92 and NK-92/5.28.z (CAR-NK) cells^2^ were cultured in X‑Vivo 10 media (Lonza) supplemented with 5% heat-inactivated human plasma (German Red Cross Blood Donation Service Baden-Württemberg – Hessen, Frankfurt am Main, Germany) and 100 U/ml IL-2 (Proleukin; Novartis Pharma, Nürnberg, Germany).

**Degranulation assays**

Tumor cells were incubated at a ratio of 1:1 with CAR-NK cells in a 96-well plate with 200 µL X-Vivo 10. APC anti-human CD107a antibody H4A3 (BioLegend, Fell, Germany) was added at 1 µL and incubated for 1h at 37 °C. 5 µL of GolgiPlug / X-Vivo solution (2 µL GolgiPlug (BD Biosciences, Heidelberg, Germany) diluted in 75 µL of X-Vivo 10) was added to each well. After an incubation time of 3h at 37 °C, cells were washed, harvested, and analyzed via flow cytometry.

**Isolation and cultivation of splenocytes from mice**

Mice were euthanized via cervical dislocation. Spleens were then isolated and dissociated using the gentleMACS Octo Dissociator (Miltenyi Biotec, Bergisch Gladbach, Germany) according to the manufacturer’s protocol. Single cell suspensions were filtered through a 70 µm cell strainer and subsequently washed with Roswell Park Memorial Institute (RPMI) medium. Erythrocyte lysis was performed in 5 ml red blood cell lysis buffer (Qiagen, Hilden, Germany) for 5 min at RT. Cells were washed twice, resuspended in 5 ml RPMI, and seeded in a 6-well plate (2.5 x 10^6^ cells/well).

**Western blot**

For Western blot analyses, tumor cells were lysed with NP40 buffer. Lysates were denatured at 95 °C in Laemmli buffer containing SDS, β-Mercaptoethanol and dithiothreitol (DTT). Proteins were separated via SDS-PAGE in a 12% TGX stain-free gel (BioRad, Feldkirchen, Germany) and transferred onto nitrocellulose membranes using a semi-dry blot system (BioRad, Feldkirchen, Germany). Human HER2 was detected with a mouse anti-human ErbB2 (HER2) antibody MJD2 (eBioscience). A secondary anti-mouse antibody was used for detection. Prior to chemiluminescence detection via ChemiDoc XRS+ (BioRad, Feldkirchen, Germany) membranes were incubated for 2 min with Weststar nC 2.0 chemiluminescent substrate (7Bioscience GmbH, Neuenburg, Germany). Analysis was performed via Image Lab software (BioRad, Feldkirchen, Germany).

**DNA and RNA extraction and quantitative real-time PCR**

DNA or RNA were extracted either from cell culture tumor cells or *ex vivo* tumor cells using either QIAamp DNA mini kit (Qiagen, Hilden, Germany) or EXTRACTME RNA isolation kit (Blirt, Gdansk, Poland) according to the manufacturer’s protocol. CDNA was synthesized using the Vilo cDNA synthesis kit (Invitrogen, Carlsbad, CA, USA). Quantitative real-time PCR (qPCR) was performed using the Absolute Blue SYBR Green Fluorescein qPCR Mastermix (Thermo Fisher Scientific, Dreieich, Germany) or FastStart Taqman® Probe Master (Sigma Aldrich, Taufkirchen, Germany). Relative gene expression levels were calculated using the ΔΔCT method.

**Murine glioblastoma models**

All *in vivo* experiments were carried out in accordance with the guidelines and regulations of the German animal protection law upon approval by the competent authority (Regierungspräsidium Darmstadt, Darmstadt, Germany, approval number FK-1088). For *in vivo* experiments, 6-8-week-old female C57BL/6 mice (Charles River Laboratories, Sulzfeld, Germany) were used. Humane endpoints were established prior to initiation of the experiments and mice were sacrificed as soon as first symptoms were observed. For orthotopic experiments animals were randomized conditional on their tumor size measured by MRI, and only mice with unequivocal tumor engraftment were included. Treatment allocation was blinded to the animal caretakers. To minimize potential confounders, cages were kept in the same room of the animal facility under SPF conditions and treatment of all cohorts was performed at the same days. In line with 3R guidelines, cage enrichments were employed. No adverse events were observed during the experiments.

Intracranial tumor cell injection was performed using a 10 μL Hamilton syringe (Hamilton, Bonaduz, Switzerland) and a Quintessential Stereotaxic Injector (Stoelting, Wood Dale, IL, USA) through a burr hole in the skull. The burr hole was located 2 mm to the right and 1 mm to the front relative to the bregma. Tumor cells were injected into the right striatum with a depth of 3 mm from the surface of the skull, at a speed of 0.5 μL/min.

After 2 min, the needle was withdrawn at a speed of 1 mm/min. Tumor growth, body weight and overall health status of all animals were monitored throughout the study. For the orthotopic model, tumor growth was measured by magnetic resonance imaging (MRI) as described below.

**Multispectral imaging**

Explanted GL261/HER2 tumors were embedded in paraffin. Next, FFPE sections were stained via Opal Polaris 7 color kit (NEL861001KT; Akoya Biosciences Inc., Marlborough, MA) against murine CD4, CD8, CD20, PD-1, Iba-1 and TMEM-119; Polaris 3 color stainings targeted murine CD3, CD4 and NK1.1; staining was performed using LabSat™ Research Automated Staining Instrument (Lunaphore Technologies SA, Tolochenaz, Switzerland). Nucleus detection was done via DAPI staining. Acquisition of stained sections were captured by Vectra Polaris instrument (Akoya Biosciences Inc.). Further, analysis was performed with (I) Phenochart®, version 1.0.12, a whole slide scan viewer, and (II) HALO™ software (Indica Labs, Albuquerque, NM), which was used for fusing of batched images to a multispectrally unmixed whole slide image and analysis.

**Highplex sequential immunofluorescence**

FFPE sections of glioma patients pre- and post-therapy were deparaffinized and rehydrated applying the standard procedure. Antigen retrieval was performed using Epredia Dewax and HIER buffer at pH 9 (Lunaphore Technologies SA) and PT Module^TM^ (Epredia) for 60’ at 102°C. The 25plex sequential-IF staining and imaging were performed using the COMET^TM^ PA platform (Lunaphore Technologies SA). The staining panel included following primary antibodies: αSMA (1:500, M0851, Dako Agilent), CD3 (1:150, A0452, DAKO Agilent), CD4 (1:50, MA5-26657, ThermoFisher Scientific), CD8 (1:100, M7103, DAKO Agilent), CD20 (1:300, M0755, DAKO Agilent), CD38 (1:50, GTX01959, GeneTex), CD68 (1:50, M0876, DAKO Agilent), CD74 (1:120, ab9514, Abcam), CD161 (1:100, ab302564, Abcam), CD163 ((1:700, ab265592, Abcam), FoxP3 (1:50, MA5-16365, ThermoFisher Scientific), GFAP (1:2500, Z0334, DAKO Agilent), GrzB (1:150, ab208586, Abcam), HER-2 (1:250, A0485, DAKO Agilent), HIF1α (1:100, NB100-134, Novus Biologicals), IL-10 (1:50, GTX632359, GeneTex), IFNγ (1:300, ab231036, Abcam), Ki-67 (1:40, M7240. DAKO Agilent), Lamp-3 (1:500, ab271053, Abcam), MPO (1:700, A039829-2, DAKO Agilent), PD1 (1:200, ab137132, Abcam), PD-L1 (1:500, ab228415, Abcam), TGFß (1:100, ab215715, Abcam), vWF (1:120, A0082, DAKO Agilent), VISTA (1:300, ab230950, Abcam).

Following anti-mouse and anti-rabbit secondary antibodies were applied: Alexa Fluor Plus 555- (1:100, 32732 or 32727, ThermoFisher Scientific) and 647-conjugated (1:200, 32733 or 32728, ThermoFisher Scientific). DAPI stain (4´6-diamidino-2-phenylindole, 1:500, 62248, ThermoFisher Scientific) was used for nuclear detection.

Image analysis was performed using the HALO digital image analysis software (Indica Labs, Inc.).

**Cytokine measurements**

Cytokine concentrations in cell culture supernatants were measured via LEGENDplex™ Inflammation Panel (Mouse) Kit (BioLegend, Fell, Germany) according to the manufacturer’s protocol. Briefly, diluted samples were mixed with beads in a V-bottom plate. The plate was incubated at 800 rpm on a plate shaker for 2h at room temperature (RT). After washing, detection antibodies were added to each well. Samples were incubated at 800 rpm on a plate shaker for 1h at RT and 25 µL of Streptavidin R-Phycoerythrin conjugate (SA-PE) were directly added to each well. After 30 min, the plate was washed again before samples were analyzed in 150 µl of washing buffer on a flow cytometer.

**NanoString sequencing**

NanoString sequencing was performed on micro-dissected GL261/HER2 tumors from FFPE samples. RNA was isolated specifically from tumor regions within FFPE sections. For gene detection nCounter PanCancer Immune Profiling Panel was used according to the manufacturer’s protocol. Further analysis of data was performed by nCounter Advanced Analysis Software and R.

**MR imaging**

Tumor engraftment was verified 7 days after tumor cell injection via a 7 Tesla Small Animal MR Scanner (Pharmascan, Bruker, Berlin, Germany). Mice were injected i.p. with 150 µl contrast agent (Gadovist, Bayer, Leverkusen, Germany), and then anesthetized with isoflurane (1.5%). During scan acquisition, the respiration rate was permanently monitored. Image acquisition was performed using Paravision 6.0.1 software in coronal planes (parameters: FOV=20x20 mm, 11 slices, 0.5 mm slice thickness, acquisition matrix = 256x256, flip angle 90°).

**Transwell assays**

Cytokine release as well as PD-1 and PD-L1 expression on tumor and immune cells was determined via transwell cytotoxicity assays using ThinCert™ cell culture inserts (Greiner Bio-One, Frickenhausen, Germany). Tumor cells were labelled with PKH67 (Sigma Aldrich) and seeded into the wells of a 6-well plate at a density of 1x10^6^ cells per well. Splenocytes were added at a density of 2.5 x 10^6^ cells per well. A cytotoxicity assay was performed in the insert for 24 h, subsequently the insert was removed. Stimulated cells in the base were cultivated for another 24 h. Cells were harvested and analyzed via flow cytometry; the supernatant was stored at -80 °C for cytokine analysis.

**Cytotoxicity assays**

Cytolytic activity of CAR-NK cells was determined using fluorescence-activated cell scanning (FACS) as previously described.^3^ Calcein violet AM (Life Technologies, Darmstadt, Germany) or PKH67 (Sigma Aldrich) labelled tumor cells were washed and co-cultured with effector cells at different effector-to-target (E/T) ratios for 2 h at 37 °C. Prior to analysis, cells were washed and resuspended in 300 μl of propidium iodide (PI; 1 μg/ml) solution. Calcein violet AM or PKH67 and PI double positive cells represented dead target cells. Spontaneous target cell lysis in the absence of effector cells was subtracted from the values to determine specific lysis.

**Schematics**

Schematics were created using BioRender.com and Adobe Illustrator.

**Supplementary Figures**

**Supplementary Figure 1:**


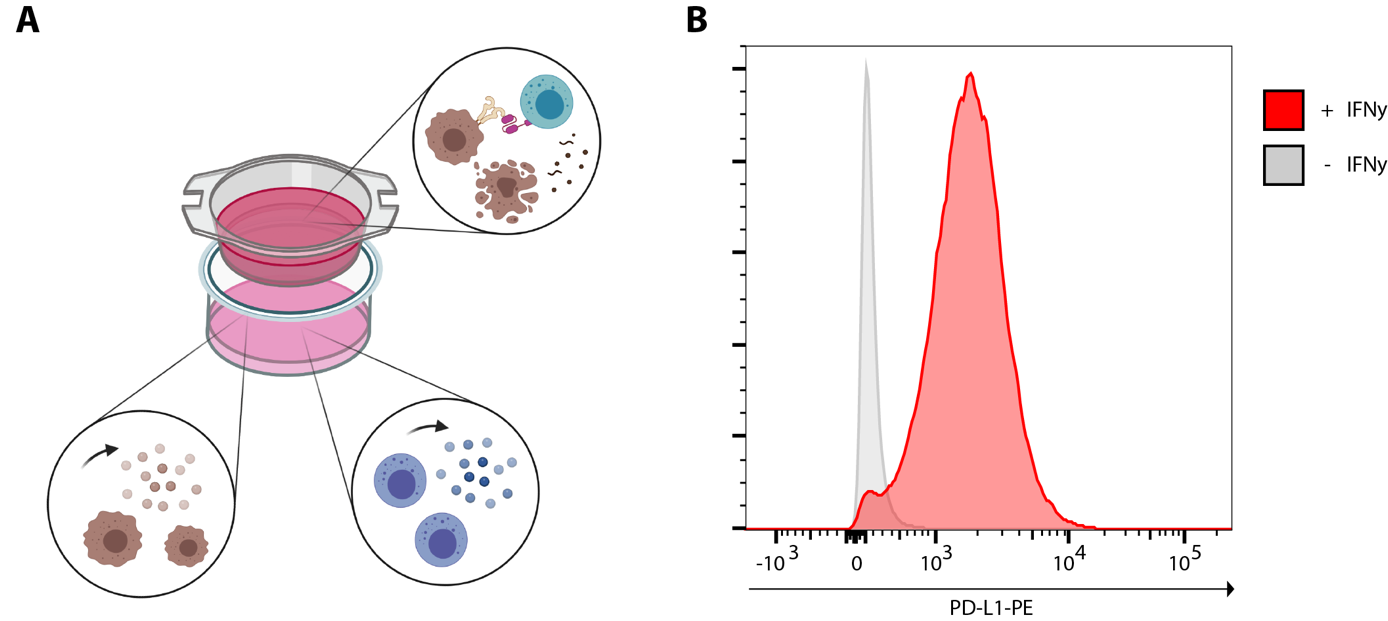


**Sup. Fig. 1: NK-92/5.28.z cell mediated lysis of HER2 positive glioblastoma cells induces PD-L1 expression. (A)** Schematic experimental setup of a co-incubation assay. In the bottom well GL261/HER2 glioma cells were co-incubated with splenocytes. An extra insert was added to the well and GL261/HER2 cells were co-incubated with or without NK-92/5.28.z CAR-NK cells. **(B)** PD-L1 staining and flow cytometry measurement of GL261/HER2 cells following preincubation of IFNγ [representative experiment shown].

**Supplementary Figure 2:**

**
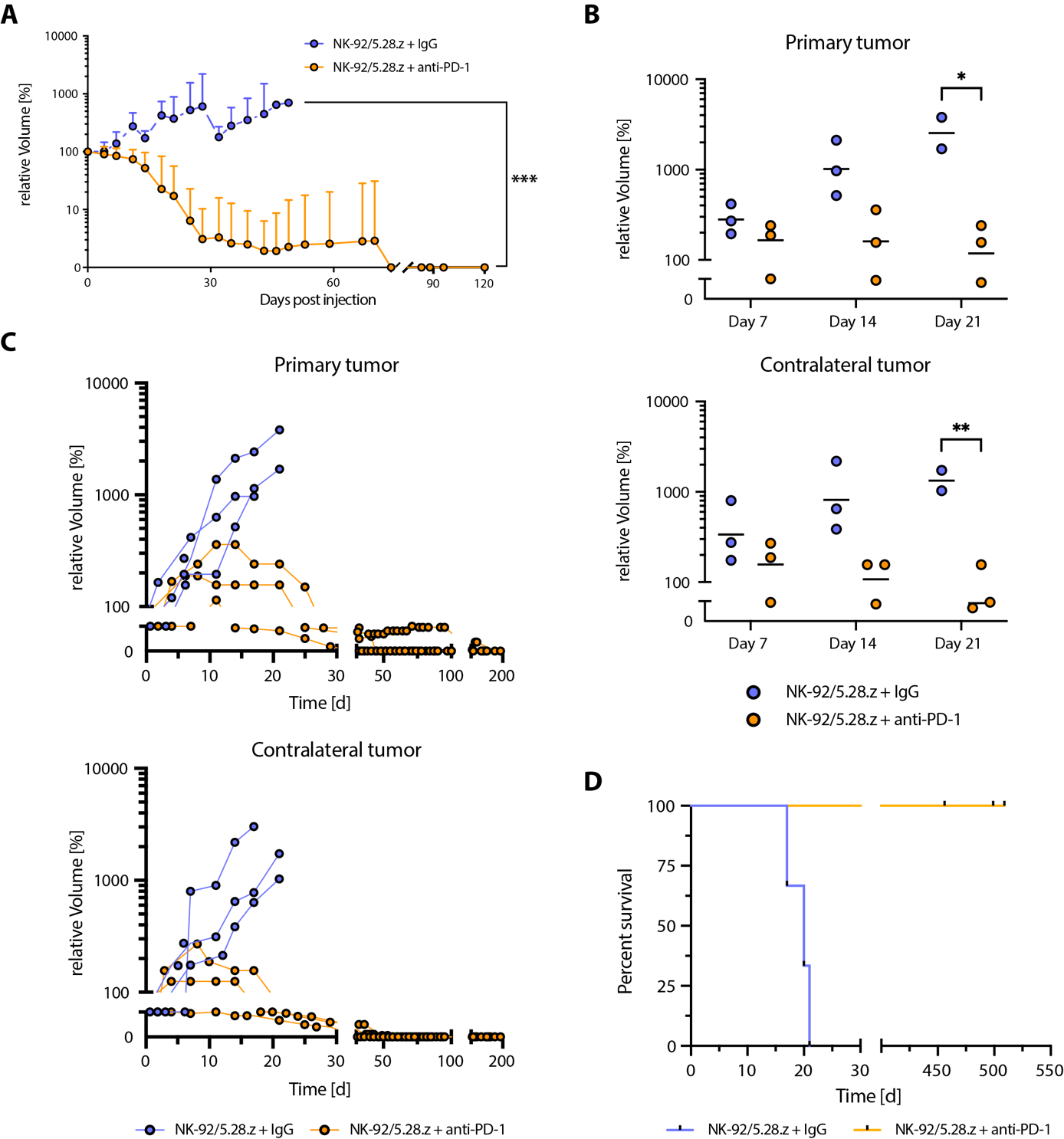
**

**Sup. Fig. 2: Efficacy of combination therapy with NK-92/5.28.z cells and anti-PD-1 antibody in the syngeneic subcutaneous GL261/HER2 tumor model. (A)** Comparison of NK-92/5.28.z monotherapy and combination therapy with anti-PD-1 antibody in mice bearing single subcutaneous tumors (n = 5-10, geometric mean ± geometric SD; two-tailed t-test with Welch correction. *** p < 0.0001). **(B-D)** Analysis of tumor growth and survival of mice bearing primary and contralateral tumor for investigation of abscopal effects. **(B)** Comparison of relative tumor growth in primary and contralateral tumors at week 1, 2 and 3 (n = 3, scatter dot blot and mean volume, each point indicates one mouse; one-tailed student’s t-test. * p < 0.05 ** p < 0.01). **(C)** Spider plot of relative tumor growth of different treatment groups **(D)** Kaplan-Meier survival analysis of the mice with bilateral tumors (n = 3, Log-Rank (Mantel-Cox) test. * p < 0.05).

**Supplementary Figure 3:**


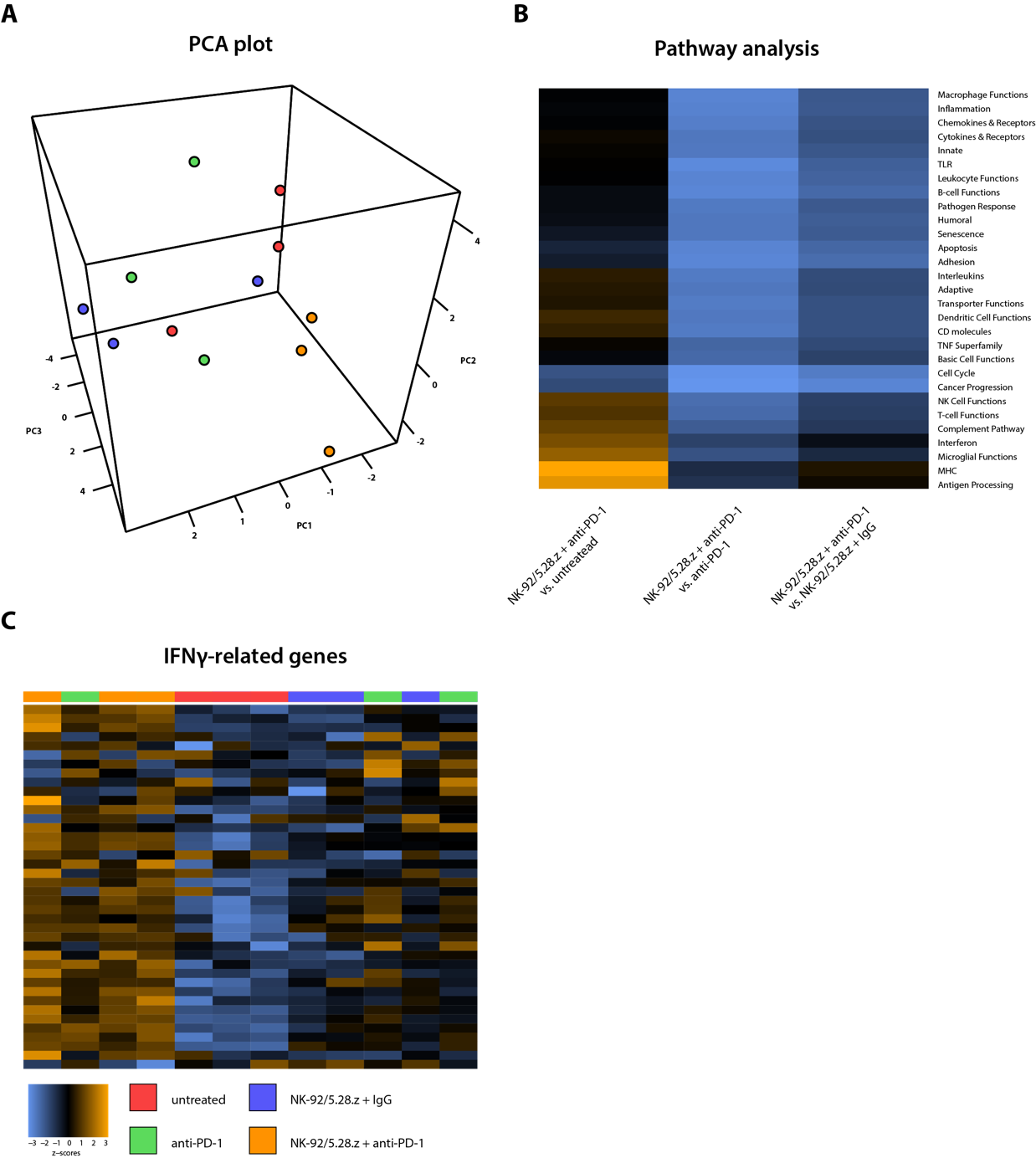


**Sup. Fig. 3: Modification of tumor immune microenvironment composition induced by combined NK‑92/5.28.z cells and anti-PD-1 checkpoint inhibition. (A-C)** Analyses of FFPE samples of orthotopic tumors with NanoString RNAseq data with comparison of differentially expressed genes after NK-92/5.28.z and anti-PD-1 combination therapy against all other treatment regimens in **(A)** 3D PCA plot. **(B)** Pathway analysis. **(C)** Heatmap of differentially expressed genes associated with IFN pathway.

**Supplementary Figure 4:**


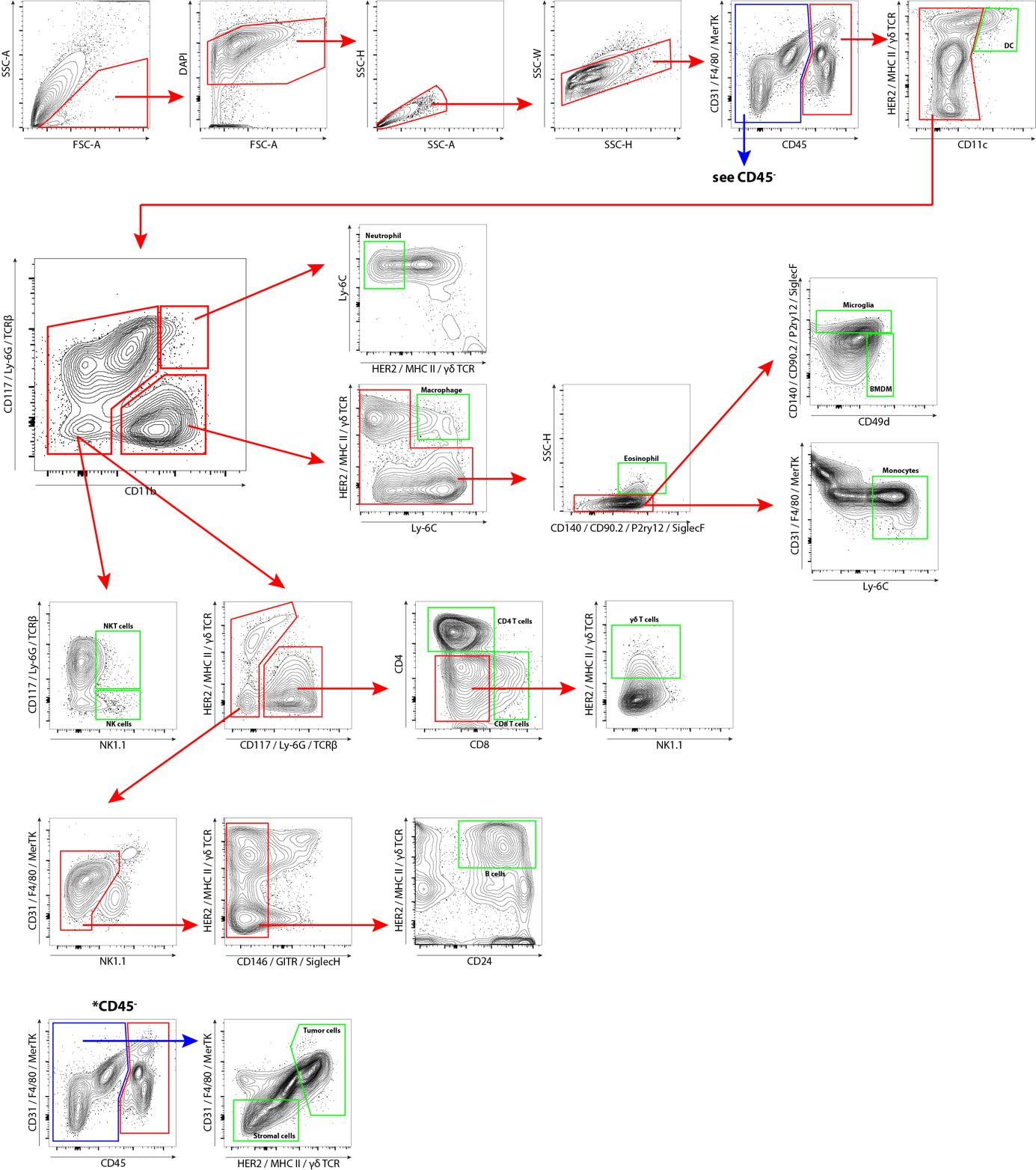


**Sup. Fig. 4: Gating strategy of high-plex extracellular brain flow cytometry panel.** To overcome instrument limitations and increase total cell surface marker detection some fluorochromes were used multiple times. Exclusive cell surface protein expression on different cells stained with the same fluorochromes was verified using FMO controls.

**Supplementary Figure 5:**


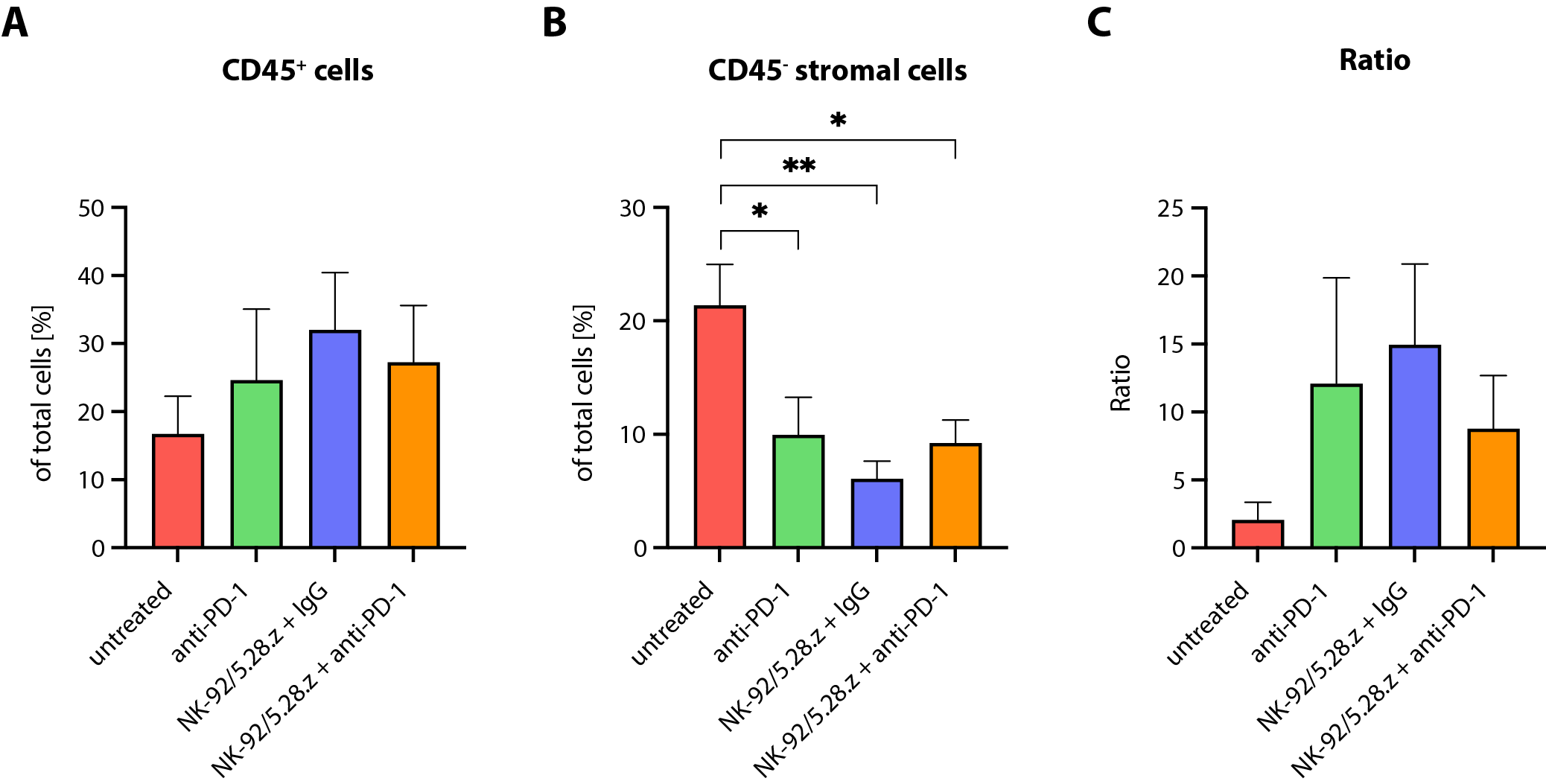


**Sup. Fig. 5: Modification of the tumor immune microenvironment induced by combination treatment with NK-92/5.28.z cells and anti-PD-1 checkpoint inhibition. (A)** Flow cytometry detection of CD45^+^ leukocytes after therapy (n = 9-11, mean + SEM). **(B)** Flow cytometry detection of CD45^-^ stromal cells after therapy (n = 9-11, mean + SEM; ANOVA with Tukey’s multiple comparison test. * p < 0.05 ** p < 0.01). **(C)** Ratio of CD45^+^ leukocytes and CD45^-^ stromal cells after therapy (n = 9-11, mean + SEM).

**Supplementary Figure 6:**

**
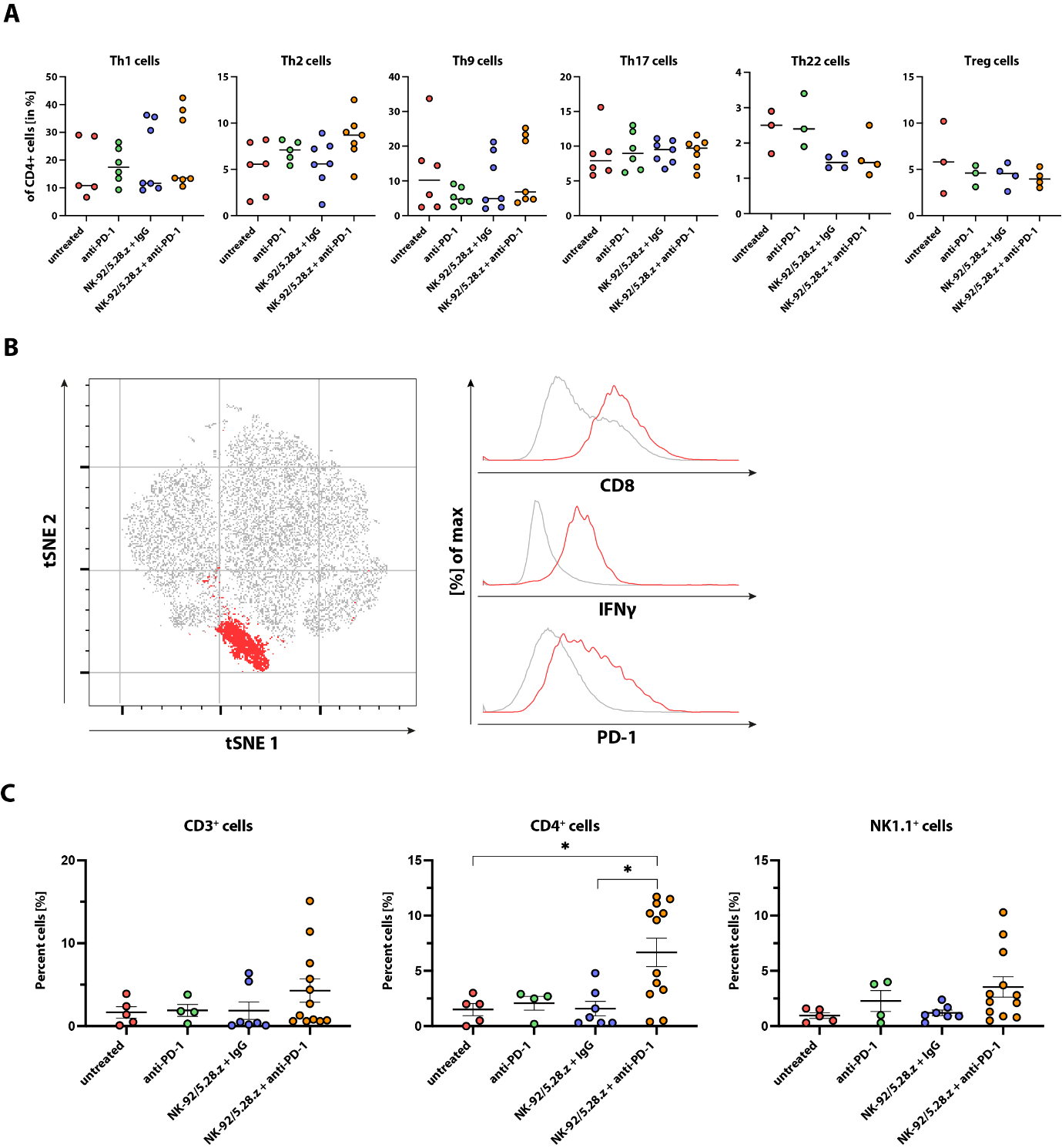
**

**Sup. Fig. 6: Superior treatment effects of NK-92/5.28.z cells and anti-PD-1 checkpoint inhibition are characterized via a CD4^+^ T cell immune response. (A)** Scatter blot of all major CD4^+^ T cell subsets detected after intracellular flow cytometry. **(B)** Expression pattern of cluster 12 regarding CD8, IFN𝛾 and PD-1. **(C)** IHC stainings of FFPE slides and scatter blot quantification of cells positive for CD3, CD4 or NK1.1 (n = 4-12, mean ± SEM, ANOVA with Tukey’s multiple comparison test. * p-value < 0.05).

**Supplementary Figure 7:**

**
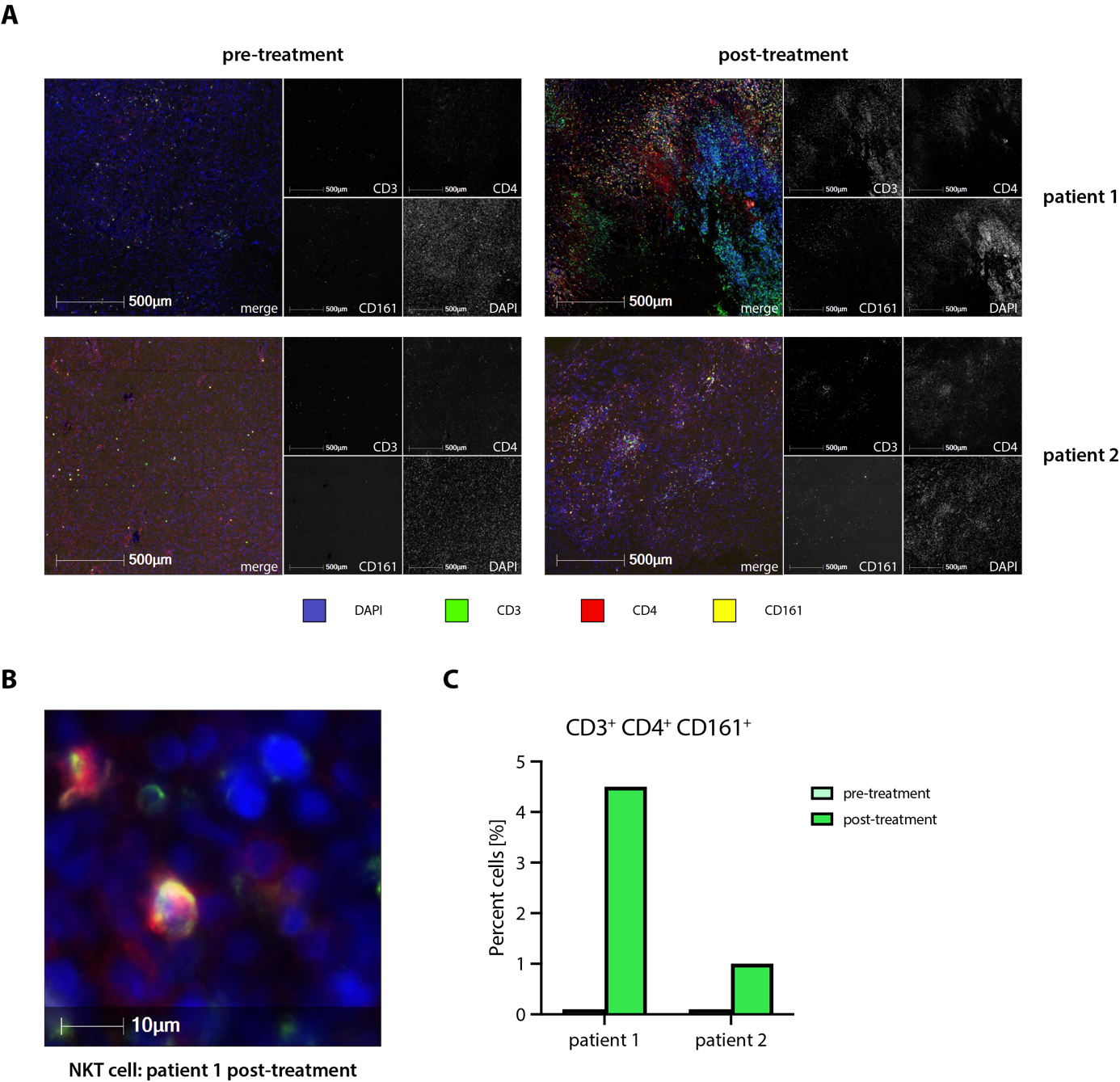
**

**Sup. Fig. 7: Increase of CD3^+^CD4^+^CD161^+^ NKT cells after combination therapy with NK-92/5.28.z cells and anti-PD-1 checkpoint inhibition. (A-C)** Analyses of FFPE samples from both CAR2BRAIN study patients where tissue pairs (pre- and post-therapy) were evaluable. **(A)** Representative stainings from both patients directly before and after combination therapy demonstrating an increase in NKT cells. Samples were stained for CD3, CD4 and CD161 to detect NKT cell infiltration. Single stainings and merged images are shown. **(B)** Representative staining from post-treatment tissue depicting a CD3^+^CD4^+^CD161^+^ NKT cell **(C)** Quantification of CD3^+^CD4^+^CD161^+^ cells from respective stainings shown in A.

**Supplementary Tables**

**Supplementary Table 1:**

| ***Antibody*** | ***Fluorochrome*** | ***Supplier*** |
| --- | --- | --- |
|  |  |  |
| CD16/CD32 | - (Fc block) | BD Bioscience |
| CD45 | APC/Fire750 | BioLegend |
| CD3 | PE | BioLegend |
| CD4 | PB | BioLegend |
| CD8 | APC | BioLegend |
| CD279 (PD-1) | FITC | BioLegend |
| CD274 (PD-L1) | PE | BioLegend |
| CD107a | APC | BioLegend |
| HER2 (anti-human) | Alexa647 | BioLegend |
| CD117 | APC-eFluor780 | eBioscience |
| CD11b | BV605 | BioLegend |
| CD11c | BV711 | BD Biosciences |
| CD140 | PE | BD Biosciences |
| CD146 | AlexaFluor488 | BD Biosciences |
| CD160 | BB700 | BD Biosciences |
| CD223 (LAG-3) | BUV737 | BD Biosciences |
| CD24 | PE-CF594 | BD Biosciences |
| CD244 | BUV805 | BD Biosciences |
| CD273 (PD-L2) | BV750 | BD Biosciences |
| CD274 (PD-L1) | BUV615 | BD Biosciences |
| CD279 (PD-1) | BV786 | BD Biosciences |
| CD31 | PE-Cy7 | eBioscience |
| CD366 (Tim-3) | BV650 | BD Biosciences |
| CD4 | BB630 | BD Biosciences |
| CD44 | PE-Cy5 | BD Biosciences |
| CD45 | AlexaFluor700 | BioLegend |
| CD49d | BV421 | BD Biosciences |
| CD8 | BUV395 | BD Biosciences |
| CD90.2 | PE | Miltenyi |
| F4/80 | PE-Cy7 | BioLegend |
| GITR | FITC | BioLegend |
| Her2 | AlexaFluor 647 | BioLegend |
| Ly-6C | BB790 | BD Biosciences |
| Ly-6G | APC-Cy7 | BioLegend |
| MerTK | PE-Cy7 | eBioscience |
| MHC II | APC | BioLegend |
| NK1.1 | BV480 | BD Biosciences |
| P2ry12 | PE | BioLegend |
| Siglec F | PE | Miltenyi |
| SiglecH | FITC | BioLegend |
| TCRß | APC-Cy7 | BioLegend |
| γδ TCR | APC | BioLegend |
| Ly-6G | APC-Cy7 | BioLegend |
| MerTK | PE-Cy7 | eBioscience |
| MHC II | APC | BioLegend |
| NK1.1 | BV480 | BD Biosciences |
| P2ry12 | PE | BioLegend |
| Siglec F | PE | Miltenyi |
| SiglecH | FITC | BioLegend |
| TCRß | APC-Cy7 | BioLegend |
| γδ TCR | APC | BioLegend |
| CD183 (CXCR3) | PE/Dazzle594 | BioLegend |
| CD185 (CXCR5) | BV650 | BD Biosciences |
| CD25 | BV510 | BioLegend |
| CD8 | BUV615 | BD Biosciences |
| FoxP3 | PE | eBioscience |
| IFN-γ | BV421 | BD Biosciences |
| IL-17A | BUV395 | BD Biosciences |
| IL-22 | PerCP/Cy5.5 | BioLegend |
| IL-33R (ST2) | BV605 | BD Biosciences |
| IL-4 | PE/Cy7 | BioLegend |
| IL-9 | APC | BioLegend |
| Ki-67 | BUV737 | BD Biosciences |

**Sup. Tab. 1: Full list of antibodies used in the experiments.** If not otherwise indicated all antibodies used are targeted against mouse. Respective fluorochrome and supplier are added to the respective antibodies.

**Supplementary Table 2:**

| **Cluster** | **Treatment group [in % of total cluster]** | | | |
| --- | --- | --- | --- | --- |
|  | *untreated* | *anti-PD-1* | *NK-92/5.28.z + IgG* | *NK-92/5.28.z + anti-PD-1* |
|  |  |  |  |  |
| ***1*** | 15,9% | 26,8% | 22,7% | 34,6% |
| ***2*** | 42,9% | 11,7% | 22,4% | 23,0% |
| ***3*** | 20,6% | 12,2% | 29,7% | 37,5% |
| ***9*** | 18,3% | 8,1% | 38,6% | 35,0% |
| ***11*** | 38,8% | 19,6% | 22,4% | 19,2% |
| ***12*** | 41,9% | 18,4% | 19,6% | 20,0% |
| ***13*** | 7,6% | 6,5% | 57,2% | 28,7% |

**Sup. Tab. 2: Phenograph analysis.** Distribution of cells according to their respective treatment group in different cluster of interest identified via Phenograph.

1. Strecker MI, Wlotzka K, Strassheimer F, Roller B, Ludmirski G, König S, et al. AAV-mediated gene transfer of a checkpoint inhibitor in combination with HER2-targeted CAR-NK cells as experimental therapy for glioblastoma. Oncoimmunology. 2022;11(1).

2. Schönfeld K, Sahm C, Zhang C, Naundorf S, Brendel C, Odendahl M, et al. Selective inhibition of tumor growth by clonal NK cells expressing an ErbB2/HER2-specific chimeric antigen receptor. Mol Ther. 2015 Feb 3;23(2):330–8.

3. Sahm C, Schönfeld K, Wels WS. Expression of IL-15 in NK cells results in rapid enrichment and selective cytotoxicity of gene-modified effectors that carry a tumor-specific antigen receptor. Cancer Immunol Immunother. 2012 Sep;61(9):1451–61.
